# Conducting Performance-Assisted Resections in the Right Temporo-Insular Cortex: A Real-Time Neuropsychological Testing (RTNT) Protocol

**DOI:** 10.3390/brainsci15090949

**Published:** 2025-08-30

**Authors:** Barbara Tomasino, Ilaria Guarracino, Tamara Ius, Miran Skrap

**Affiliations:** 1 Scientific Institute, IRCCS E. Medea, Dipartimento/Unità Operativa Pasian di Prato, 33037 Pasian di Prato, Italy; ilaria.guarracino@lanostrafamiglia.it; 2 Academic Neurosurgery, Department of Neuroscience, University of Padova, 35121 Padova, Italy; tamara.ius@aoud.sanita.fvg.it; 3 Neurosurgery Unit, Head-Neck and Neurosciences Department, Azienda Ospedaliero Universitaria Friuli Centrale, 33100 Udine, Italy; skrap@aoud.sanita.fvg.it

**Keywords:** neuropsychology, glioma, temporal lobe, awake surgery

## Abstract

Background/Objectives: There is increasing interest within cognitive neuro-surgery in preserving domains not traditionally assessed during awake surgery. The study aims at proposing a specific protocol to assist surgical resection in right temporal areas. Patients were not evaluated during direct cortical stimulation; instead, assessments occurred during the resection itself. The real-time neuropsychological testing (RTNT) protocol employed tasks evaluating visuospatial and social cognition, administered repeatedly throughout the resection using varied items. Methods: A consecutive series of 24 patients (median age 44) performed RTNT. The aim of RTNT is to maintain high accuracy through resection. Lesions in the right temporal cortex and the subcortical white matter beneath can cause deficits; accordingly, not all of our patients had pre-surgery performance within the normal range. In this case, the aim of RTNT is to maintain the not perfect pre-surgery level. Results: We found a statistically significant between-tasks difference in the patients’ median values (across RTNT runs), in their minimum score reached during resection, and in the delta between performance at the last vs. the first RTNT run. The tasks that varied belonged to visual–spatial attention (landmark task), face processing (recognition of famous faces), and social cognition (theory of mind). The outcome was measured by pre- vs. post-surgery neuropsychological score comparison. The number of patients scoring below the normal range did not significantly differ between post- vs. pre-intervention. Conclusions: Results demonstrated the feasibility of implementing a continuous monitoring protocol during the resection phase, and the potential of the selected tasks to assess visuospatial and social functions associated with the non-dominant (right) hemisphere.

## 1. Introduction

Awake surgery is an effective method to maximize surgical resection of brain tumors while minimizing the risk of cognitive sequelae [1]. Nowadays, awake surgery aims at also monitoring extra-language functions [2,3] whose deficits would have important effects on the patients’ quality of life [4]. Extra-language functions include visual–spatial functions such as perception, spatial representation, or spatial awareness, as well as functions related to social cognition, such as theory of mind, empathy, and non-verbal language, supported by the right hemisphere. The literature reports about direct cortical stimulation mapping (DES) in the right temporo-parietal lobe while administering a barrage task, with evidence for significant spatial deviations to the right side following inactivation of the supramarginal gyrus, the caudal portion of the superior temporal gyrus, and at the subcortical level of the superior occipitofrontal fascicle [5]. Reviews of the literature on awake surgery [3,6] identified which are the commonly used tasks for DES in the right hemisphere. They are the line bisection or the cancelation tasks to test visuospatial cognition, the Ekman’s faces task for testing face emotion recognition, and the “reading the mind in the eyes” or the false-belief task for testing social cognition. As for the DES positive sites, it emerges that spatial and social cognition are the domains to be tested for surgeries in the right temporal cortex [7].

Despite the wealth of neuropsychological tests mentioned in the reviews above and the emerging need to monitor as many cognitive functions as possible, none of the above study presents a neuropsychological protocol as they report the usefulness of single tests, e.g., line bisection; second, all the studies but one [8], performed a task during DES, leaving open the possibility to perform cognitive monitoring during resection interleaved with DES. In this study, we present a real-time neuropsychological testing (RTNT, [9]) protocol for resections in the right temporo-insular area. RTNT protocols for other areas [10,11,12,13] have been proven to be feasible.

The RTNT has been designed [9] to assist surgery, and it is an intensive neuropsychological monitoring method complementary to DES. It starts with the beginning of resection and it ends with the beginning of hemostasis. The aim is to have continuous feedback on the patient’s cognitive status during resection. Accordingly, in the present study, the aim is to obtain as much as possible a complete overview of the neuropsychological functions during surgery in the right temporo-insular area and to determine whether and how the neuropsychological status evolves during resection. Other authors used the RTNT approach [8]: they report a single case of a patient who performed RTNT during resection for a recurrent right temporal astrocytoma. We report data on a consecutive series of 24 patients who performed RTNT and comment on the clinical implications of their pattern of results.

## 2. Materials and Methods

### 2.1. Participants

We filtered from our database a consecutive series of adult patients who were retrospectively reviewed for the purpose of the present study. The study was approved by the Ethics Committee (0004890/P/GEN/ARCS, ID 4202) and carried out in accordance with the 2013 Fortaleza version of the Helsinki Declaration and subsequent amendments. As the study was retrospective, written consent to participate in the study was not applicable. Written informed consent was obtained for surgery.

### 2.2. Inclusion Criteria

Inclusion criteria were as follows: planned surgical removal between 2011 and 2021 for right-hemisphere tumor or cavernoma involving the temporal or temporo-insular area; having performed the real-time neuropsychological testing during resection; native Italian speakers; normal or corrected-to-normal vision.

### 2.3. Exclusion Criteria

Exclusion criteria were history of psychiatric or drug abuse, developmental language problems or learning disabilities, or a family history of such disabilities.

### 2.4. RTNT Protocol

**Figure 1:**
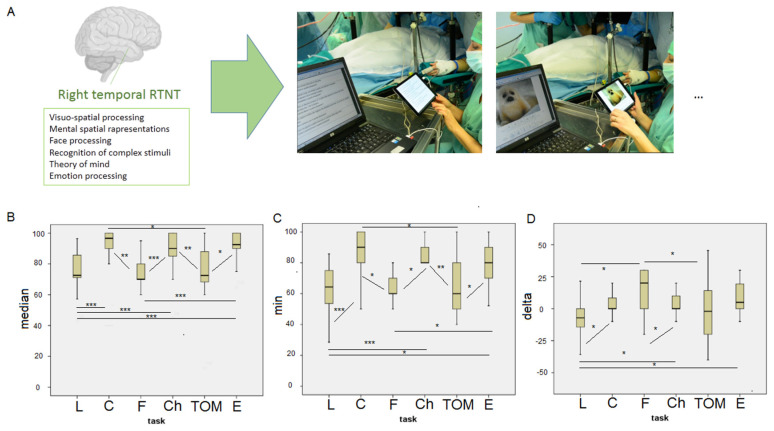
The domains assessed by the right temporal RTNT protocol (**A**) along with the median (**B**), minimum (**C**) scores and the delta between the last and the first RTNT run (**D**). Significant differences are marked as *p* < 0.001 ***; *p* < 0.005 **, and *p* < 0.01 *.

RTNT is administered whilst the surgical resection proceeds, and it is alternated with DES. Its duration can therefore vary, and it depends on the length of the resection itself. The duration range in the present series was 28 min to 76 min. The RTNT is composed of a variable number of runs, each of which is composed of a fixed series of tests that rapidly measure a specific cognitive domain considered important for that anatomical location. Each test is made up of 10 items. The administration therefore requires that the patient respond to all 10 items for each test, then continues with performing the next test (see Table 1 and Figure 1A for the protocol of the test used). Each test performance is compared to the pre-surgery level (RTNT uses percentages, 100% being optimal brain function). If altered performances in one or more domains were identified in the pre-intervention assessment, these are indicated on the RTNT protocol in order to understand whether any decrease from 100% is to be considered in line with the patient’s pre-performance or it is a signal of a decrease to be monitored [9]. The RTNT protocol for right temporal resections was constructed by including tasks related to cognitive domains that, from the literature, are of relevance to the right hemisphere. We considered both functions supported by the temporal cortex (e.g., temporo-parietal junction for visuospatial cognition [14,15], right part of the superior temporal sulcus for facial emotion recognition [16]; temporo-parietal junction for empathy and TOM) and functions supported by areas connected to it via subcortical white matter (e.g., Superior Longitudinal Fasciculus, Inferior Frontal Occipital Fasciculus, Inferior Longitudinal Fasciculus, Uncinate Fasciculus [17,18]).

### 2.5. Pre- and Post-Surgery Neuropsychological Assessment

All patients performed a neuropsychological assessment before and after (one week) surgery. The tests are site-specific in order to have a picture of the cognitive functioning of the patient for the domains concerned. Normal range is defined according to each test’s normative data. Raven’s colored progressive matrices [25] measured fluid intelligence; Trail making test [26], Corsi span backward and forward [27] and Symbol Digit Modalities Test [28] measured executive functions and attention; Rey–Osterrieth complex figure [29] and face recognition [21,30] tested visuospatial memory and semantic memory; constructive apraxia [31], behavioral inattention test (reading; [32]), landmark task [24], imaginative and perceptual battery (clock test, [23]) and little man task [33] assessed visuo-constructive and visuo-perceptual processing.

## 3. Results

### 3.1. Patients

**Figure 2:**
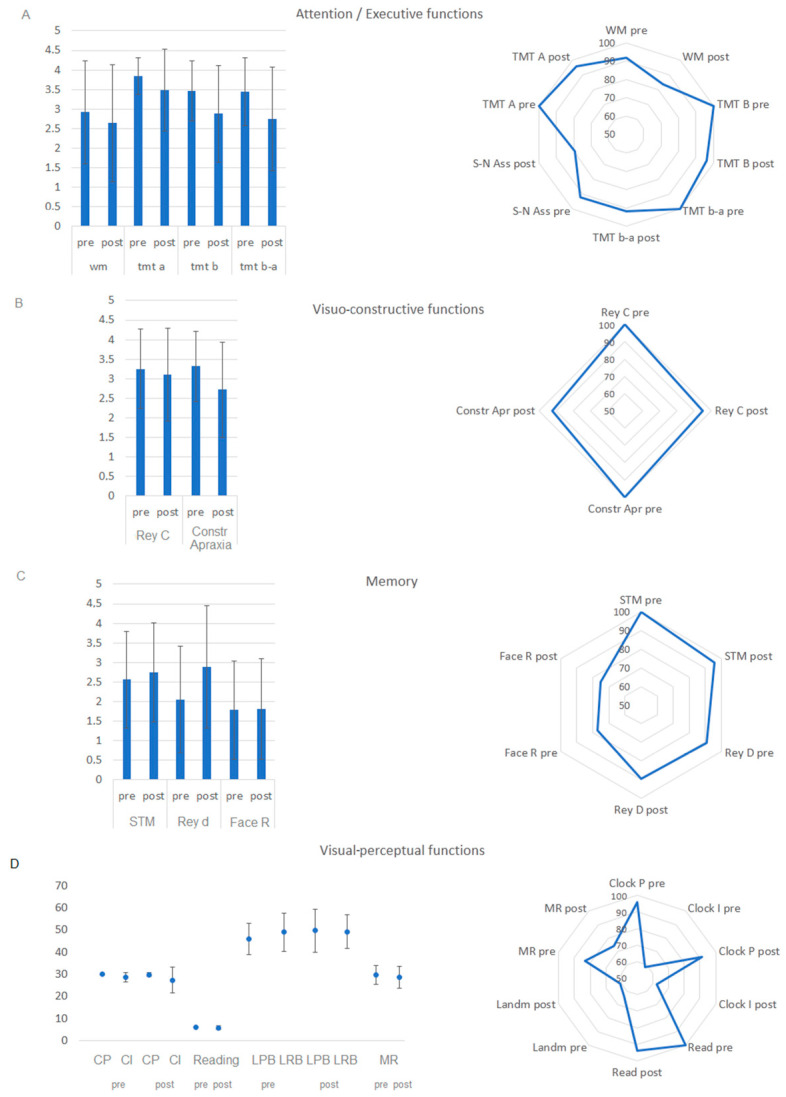
The level of performance, expressed as equivalent scores (a score laying below or equal to the external nonparametric tolerance limit of adjusted scores corresponding to 0, 1, 2, and 3 are intermediate, on the left side of the panel) and the percentage of within the normal range performances (on the right side of the panel) before and immediately post-surgery for attention/executive functions (**A**), visuo-constructive functions (**B**), memory (**C**), and visuo-perceptual functions (**D**). For the latter domain, mean performance (corrected scores are reported, see Method section).

A consecutive series of 26 right-handed adult patients was included. Two patients were excluded from the data analyses because one fell asleep at the beginning of surgery (P#2) and the other had a severe psychomotor agitation (P#14). The final group size was 24 (see Table 2 for their clinical and demographic details). At pre-surgery neuropsychological testing, all the subjects scored within the normal range at Raven’s matrices, visual–spatial short-term memory, reading, Trail making test, copy of Rey’s figure, and constructive apraxia (for their percentage accuracy see Figure 2). Fewer patients scored within the normal range at visual working memory (92%), delayed recall of the complex Rey’s figure (90%), clock test (95% at perceptual subtest and 58% at imaginative subtest), landmark test (63%), number–symbol association (92%), famous people face recognition (76%), and little man task (83%). They all underwent awake surgery and RTNT.

### 3.2. RTNT Results

A Kruskal–Wallis H test showed a statistically significant difference in the patients’ median values (across RTNT runs), minimum scores, and delta (performance at the last vs. first RTNT run) between tasks (see Figure 1B–D and Table 3). L, F, and TOM showed the greatest variation (see Table 3). For both the median values and for the minimum scores, L significantly differed from C, Ch, and E; F significantly differed from C, Ch, and E. TOM significantly differed from C, Ch, and E (see Figure 1B,C). The difference between tasks in the patients’ delta showed both improvements and decrements for L, F, and TOM. The L delta significantly differed from C, F, Ch, and E; F significantly differed from Ch; TOM significantly differed from F (see Figure 1D).

### 3.3. Intra-RTNT Qualitative Observations

In this case series, we detected the following: (i) emotion-related changes in two patients (a change in mood from a certain point on, P#11, and a high anxiety from a certain point on, asking for hand continually and shaking it, P#12); (ii) falling asleep (P#16, P#21, P#12, P#19, P#1, P# 7, P#17) and (iii) reporting pain from a certain point (P#8, P#19, P#4, P#6, P#11) when resection approached the insula and manipulation involved the vessels.

### 3.4. Neuropsychological Outcome

All patients were assessed one week after the intervention. The number of patients scoring below the normal range did not significantly differ between post- vs. pre-intervention (see Table 4). There were tests at which the number of patients scoring in the normal range remained consistently high (accuracy > 95%, visual–spatial short-term memory, Figure of Rey Copy, reading, TMT A and TMT B, and accuracy < 95% > 85: visual–spatial working memory, Rey D, clock (perceptual subtest), constructional apraxia, and TMT B-A see Figure 2) and tests at which the number of patients scoring within the normal range was lower in both pre-surgery and was maintained so in the post-surgery (accuracy < 85%: clock imaginative subtest, landmark, symbol–digit association, face recognition, and little man task; see Figure 2). As a further characterization of patients’ cognitive outcome, we also analyzed patients’ level of performance and found that this measure was significantly lower at post- vs. pre-intervention for constructive apraxia and digit symbol association, while all the other tasks showed no significant difference between post vs. pre-intervention (see Table 4 and Figure 2). As a qualitative further analysis, equivalent scores were used to check whether patients who showed a borderline performance (equivalent score 1) at pre-surgery testing improved at post-surgery assessment (equivalent scores 2–4), and this was the case for visual–spatial short-term memory and Rey Delayed Recall (see Figure 2).

## 4. Discussion

The understanding of the role of the right hemisphere in various cognitive domains has led more and more neurosurgeons to proceed with awake surgery for the resection of tumors located in the right hemisphere [2]. In a review [35], authors considered 13 papers in which speech, visuospatial cognition, executive functions, social cognition, working memory, spatial attention, and sensory–motor functions were tested during DES in the right hemisphere. The authors emphasize the importance of testing the non-dominant hemisphere in order to preserve functions whose impairment can have a major impact on the patient’s life.

In the present study, we used an RTNT protocol to assist surgery in the right temporal cortex. Of the RTNT tasks used, three revealed vulnerability to resection, namely the landmark task, famous face recognition, and theory of mind. To date, not all the patients’ pre-surgery had a performance within the normal range. The landmark task and famous people face recognition were among those. This result suggests that during surgery it is worth monitoring these functions as they are already mildly impaired pre-surgery. This is, however, a selective vulnerability as other tests for which fewer patients had pre-surgery within normal range performance were not altered during RTNT. Deficits in visuospatial processing are associated with lesions in the right temporal cortex and subcortical white matter (fronto-parietal network mainly represented by the posterior parietal cortex and its connections with the prefrontal cortex through the Superior Longitudinal Fasciculus, SLF) [36,37]. The landmark task allows for obtaining an intrasurgical score by which the patient is monitored with respect to the possible onset of spatial neglect [24]. RTNT results are also in line with the knowledge that the right temporal cortex is involved in face processing [38]. The fusiform gyrus, along with the inferior longitudinal fascicles, is part of the network involved in processing face stimuli. Lastly, the theory of mind, which corresponds to the ability to understand others’ mental states, is supported by a network of areas involving the right superior temporal lobe, along with the temporo-parietal junction, the medial prefrontal cortex, and the subcortical white matter connecting these areas. To date, the analysis of the delta between the last RTNT run and the first RTNT run showed both decreases (landmark task and theory of mind) and improvements (face recognition). Importantly, decreases were mild (lower-bound 95% CI for landmark task and theory of mind were, respectively, −19.17 and −25.82). Taken together, results suggest that the temporal RTNT protocol may be used as a tool, complementary to DES, that offers the possibility to monitor whether and how the neuropsychological status evolves during resection. The analysis of the outcome shows a non-significant pre-vs. post-surgery difference in the number of patients scoring within the normal range. The plasticity present before surgery that allows the maintenance of cognitive functioning could also be massively present after surgery, to the point of allowing a rapid recovery after few months [39]. RTNT can contribute to maintaining the patients’ neuropsychological status at the pre-surgery level. This is in line with the recommendations emerging in the different reviews of the literature on awake surgery for extra-language functions, suggesting that it is worth monitoring also non-dominant hemisphere-related functions in order to avoid post-operative deficits [2,3,6].

A separate comment deserves the observation of behavioral changes that can occur during surgery. Three different behavioral changes were detected: a change in mood, falling asleep, and pain experienced from a certain point of surgery. Observing these changes allows in a larger study with more case histories to localize the anatomical correlate of these behavioral changes. Twelve patients demonstrating these behaviors now are few to allow for anatomical discussion; however, their lesion localization is consistent with the literature and the knowledge of brain circuits involved in mood changes [40], sleep [41], and pain [42].

### Limitations of the Study

We acknowledge that the main limitation of the study is that patients were not assessed with the same tests before, during, and after surgery. The protocol match was followed for same cognitive domains and not for others. We believe that this could be understandable in the context of a complex procedure and patient availability. However, the mismatch affects comparisons across phases that are not strictly equivalent to the proposed protocol.

A second limitation is represented by the sample size, which is small, and by inter-individual variability in the results; both are factors constraining the generalizability of the conclusions.

## 5. Conclusions

The study highlights the feasibility of implementing a continuous monitoring protocol during the resection phase, and the potential of the temporal RTNT to assess visuospatial and social functions. Future studies with a bigger sample size and pre-intra-post-surgery matched testing protocols are necessary to obtain a more complete patient profile.

## Figures and Tables

**Table 1:** RTNT protocol for right temporal resections.

**RTNT Test-Run**	**Test**	**Assessed Ability**
International Affective Picture System (E) [19]	The patient is presented with positive, negative, or neutral images. The patient using the SAM Manikin provides a score on the Likert scale for pleasure and arousal.	Explicit measures of emotion-related processing
Real object or chimera (Ch) [20]	Black and white images are presented corresponding to real figures (living and non-living) or chimeras (figures formed by the fusion of 2 living or 2 non-living). The patient says whether the image is real or a chimera.	Visual–perceptual and semantic skills
Face Recognition (guess of familiarity) and identification (F) [21]	Black and white photos of famous and unknown people are presented. The subject says whether the person is famous or not and if they are, the patient says their name.	Semantic memory (Familiarity Recognition Units, Personal Identity Nodes)
Theory of Mind (TOM) [22]	The subject is presented with very short scenes with a protagonist. The subject says what emotion the protagonist will feel in the specific situation described.	Ability to put oneself in the shoes of others and understand their mental states
Clock test: Imaginative (C) subtest [23]	The patient is orally told a time, e.g., 14:30, mentally pictures the clock, and says whether the hands are positioned both on the right half of the dial, both on the left half, or are one on the right and one on the left.	Spatial cognition in an imaginative dimension
Landmark test (L) [24]	Segments are presented. Each segment is made up of a red and a black portion. The subject says whether the longer portion is the red or the black one.	Highlights the presence of spatial neglect

**Table 2:** Demographic, Surgical, Radiological, and Molecular Data.

Title 1	Title 2
Number of patients	24
Sex	
Male	16
Female	8
Age (years)	
Median (years and range)	44 (66–23)
Tumor side	
Right	24
Handedness	
Right	24/24
Education	
Median (years and range)	13 (20–8)
Molecular class ^^^	
High-grade glioma	11
Oligodendrogliomas, IDH1/2 mutant 1p/19q codeleted	4
Astrocytomas, IDH1/2 mutant	6
Astrocytomas, IDH1/2 wild type	1
Cavernoma	2
Preoperative tumoral volume (cm^3^)	
Median (range)	42 (15–118)
EOR	
Median (range)	99 (34–100)
Intraoperative protocol	
Awake surgery	24/24

^^^ Based on the 2016 World Health Organization classification criteria [34].

**Table 3:** Patients’ RTNT performance during resection and significance of the between-task Kruskal–Wallis H test. The values for the task that varied most are reported in bold.

	95% CI Lower	95% CI Upper	Median	Min (Baseline: 1st RTNT Run)	Max
**Median RTNT scores (** **χ^2^ (5) = 41.02, *p* < 0.001)**					
L	63.56	80.57	**71.42**	42.85 (78.57)	89.28
C	92.65	100	100	85 (93.3)	100
F	63.69	79.94	**70**	45 (70)	90
Ch	87.91	98.44	95	80 (90)	100
TOM	68.7	87.82	**75**	60 (80)	100
E	88.59	98.81	95	75.71 (90)	100
**Minimum RTNT score (** **χ^2^ (5) = 31.23, *p* < 0.001)**					
L	**42.24**	67.73	57.14	28.57	78.57
C	85.94	97.68	90	80	100
F	**49.86**	69.53	60	30	80
Ch	79.1	88.16	80	80	100
TOM	**54.62**	78.1	60	40	100
E	72.77	93.07	80	52.14	100
**Delta: last RTNT run—first RTNT run (** **χ^2^ (5) = 15.71, *p* < 0.01)**					
L	−19.17	0.99	−7.1	**−35.72**	14.29
C	−3.34	8.80	0	−10	20
F	−1.85	23.06	20	**−20**	30
Ch	−1.61	7.07	0	−10	10
TOM	−25.82	3.6	−20	**−40**	20
E	−0.26	16.36	0	−10	30

CI = Confidence Interval; L = Landmark Task; C = Clock Test; F = Face recognition; Ch = Chimera recognition; TOM = Theory of Mind; E = Emotion Processing.

**Table 4:** The Wilcoxon test for two related samples determined whether the number of performances within the normal range and the level of performance differ before vs. post-surgery. Significant differences are reported in bold.

Test	Level of Performance	Number of Within the Normal Range Performances
Short-term memory	Z = −691 *p* = 0.490	Z = −1 *p* = 0.317
Working memory	Z = −1.312 *p* = 0.190	Z = −1 *p* = 0.317
Rey Copy	Z = 0.00 *p* = 1	Z = −1 *p* = 0.317
Rey Delayed Recall	Z = −1.448 *p* = 0.148	Z = 0 *p* = 1000
Clock Perceptive	Z = −0.816 *p* = 0.414	Z = −0.577 *p* = 0.564
Clock Imagery	Z = −0.834 *p* = 0.404	Z = −0.302 *p* = 0.763
Constructional praxis	**Z = −2.712 *p* = 0.007**	Z = −1.414 *p* = 0.157
Reading	Z = −1.00 *p* = 0.317	Z = −1.00 *p* = 0.317
Landmark Perceptual Bias	Z = −0.426 *p* = 0.670	Z = −1.00 *p* = 0.317
Landmark Response Bias	Z = −0.877 *p*= 0.381	
Digit symbol association	**Z = −2.613 *p* = 0.009**	Z = −1.342 *p* = 0.180
Trail Making Test A	Z = −1.461 *p* = 0.144	Z = −1 *p* = 0.317
Trail Making Test B	Z = −1.586 *p* = 0.113	Z = −1 *p* = 0.317
Trail Making Test-B-A	Z = −1.5361 *p* = 0.124	Z = −1.414 *p* = 0.157
Face recognition	Z = −1.612 *p* = 0.107	Z = −1 *p* = 0.317
Little Man	Z = −1.944 *p* = 0.052	Z = −1.732 *p* = 0.083

## Data Availability

Data will be available upon request due to ethical reasons.

## Abbreviations

The following abbreviations are used in this manuscript:

RTNT	Real-time neuropsychological testing
DES	Direct electrical stimulation
TOM	Theory of mind
L	Landmark test
C	Clock test
Ch	Chimera test
F	Face recognition
I	Emotion processing

## References

[1] Ojemann G., Ojemann J., Lettich E., Berger M. (1989). Cortical language localization in left, dominant hemisphere. An electrical stimulation mapping investigation in 117 patients. J. Neurosurg..

[2] Vilasboas T., Herbet G., Duffau H. (2017). Challenging the Myth of Right Nondominant Hemisphere: Lessons from Corticosubcortical Stimulation Mapping in Awake Surgery and Surgical Implications. World Neurosurg..

[3] Mamadaliev D.M., Saito R., Motomura K., Ohka F., Scalia G., Umana G.E., Conti A., Chaurasia B. (2024). Awake Craniotomy for Gliomas in the Non-Dominant Right Hemisphere: A Comprehensive Review. Cancers.

[4] Duffau H. (2010). Awake surgery for nonlanguage mapping. Neurosurgery.

[5] Bartolomeo P. (2006). A parietofrontal network for spatial awareness in the right hemisphere of the human brain. Arch. Neurol..

[6] Lemée J.M., Bernard F., Ter Minassian A., Menei P. (2018). Right Hemisphere Cognitive Functions: From Clinical and Anatomical Bases to Brain Mapping During Awake Craniotomy. Part II: Neuropsychological Tasks and Brain Mapping. World Neurosurg..

[7] Martín-Monzón I., Amores-Carrera L., Sabsevitz D., Herbet G. (2024). Intraoperative mapping of the right hemisphere: A systematic review of protocols that evaluate cognitive and social cognitive functions. Front. Psychol..

[8] Gecici N.N., Habib A., Niranjan A., Balzer J., Sherry N., Zinn P.O. (2025). Optimizing brain mapping: Integrating real-time neuropsychological assessment in awake craniotomy. Neurosurg. Focus. Video.

[9] Skrap M., Marin D., Ius T., Fabbro F., Tomasino B. (2016). Brain mapping: A novel intraoperative neuropsychological approach. J. Neurosurg..

[10] Tomasino B., Guarracino I., Ius T., Maieron M., Skrap M. (2021). Real-Time Neuropsychological Testing Protocol for Left Temporal Brain Tumor Surgery: A Technical Note and Case Report. Front. Hum. Neurosci..

[11] Tomasino B., Guarracino I., Ius T., Budai R., Skrap M. (2022). Real-Time Neuropsychological Testing of Sensorimotor Cognition During Awake Surgery in Precentral and Postsomatosensory Areas. World Neurosurg..

[12] Tomasino B., Guarracino I., Ius T., Skrap M. (2023). Continuous Real-Time Neuropsychological Testing during Resection Phase in Left and Right Prefrontal Brain Tumors. Curr. Oncol..

[13] Guarracino I., Ius T., Pauletto G., Maieron M., Skrap M., Tomasino B. (2020). Junior-Real Time neuropsychological testing (j-RTNT) for a young patient undergoing awake craniotomy. Brain Cogn..

[14] Committeri G., Pitzalis S., Galati G., Patria F., Pelle G., Sabatini U., Castriota-Scanderbeg A., Piccardi L., Guariglia C., Pizzamiglio L. (2007). Neural bases of personal and extrapersonal neglect in humans. Brain J. Neurol..

[15] Rengachary J., He B.J., Shulman G.L., Corbetta M. (2011). A behavioral analysis of spatial neglect and its recovery after stroke. Front. Hum. Neurosci..

[16] Wang X., Song Y., Zhen Z., Liu J. (2016). Functional integration of the posterior superior temporal sulcus correlates with facial expression recognition. Brain Mapp..

[17] Genova H.M., Rajagopalan V., Chiaravalloti N., Binder A., Deluca J., Lengenfelder J. (2015). Facial affect recognition linked to damage in specific white matter tracts in traumatic brain injury. Soc. Neurosci..

[18] Philippi C.L., Mehta S., Grabowski T., Adolphs R., Rudrauf D. (2009). Damage to association fiber tracts impairs recognition of the facial expression of emotion. J. Neurosci..

[19] Lang P.J., Bradley M.M., Cuthbert B.N. (1997). International affective picture system (IAPS): Technical manual and affective ratings. NIMH Cent. Study Emot. Atten..

[20] Humphreys G.W., Riddoch J.M. (1993). Birmingham Object Recognition Battery.

[21] Bizzozero I., Ferrari F., Pozzoli S., Saetti M.C., Spinnler H. (2005). Who is who: Italian norms for visual recognition and identification of celebrities. Neurol. Sci..

[22] Prior M., Marchi S., Sartori G. (2003). Social Cognition and Behavior. A Tool for Assessment Cognizione Sociale e Comportamento. Uno Strumento Per la Misurazione.

[23] Antonietti A., Bartolomeo P., Colombi A., Incorpora C., Oliveri S. (2008). Batteria Immaginazione e Percezione (BIP) per la valutazione della cognizione visuo-spaziale-Italian Translation and Adaptation of the Corresponding Battery Devised by P. Bartolomeo, A. C. Bachoud-Levi and S. Chokron at the INSERM, Paris.

[24] Capitani E., Neppi-Mòdona M., Bisiach E. (2000). Verbal-response and manual-response versions of the Milner Landmark task: Normative data. Cortex.

[25] Basso A., Capitani E., Laiacona M. (1987). Raven’s coloured progressive matrices: Normative values on 305 adult normal controls. Funct. Neurol..

[26] Giovagnoli A.R., Del Pesce M., Mascheroni S., Simoncelli M., Laiacona M., Capitani E. (1996). Trail making test: Normative values from 287 normal adult controls. Ital. J. Neurol. Sci..

[27] Monaco M., Costa A., Caltagirone C., Carlesimo G.A. (2015). Erratum to: Forward and backward span for verbal and visuo-spatial data: Standardization and normative data from an Italian adult population. Neurol. Sci..

[28] Wechsler D. (1997). Wechsler Adult Intelligence Scale—Third Edition (WAIS-III).

[29] Caffarra P., Vezzadini G., Dieci F., Zonato F., Venneri A. (2002). Rey-Osterrieth complex figure: Normative values in an Italian population sample. Neurol. Sci..

[30] Bizzozero I., Lucchelli F., Pozzoli S., Saetti M.C., Spinnler H. (2007). “What do you know about Ho Chi Minh?” Italian norms of proper name comprehension. Neurol. Sci..

[31] Spinnler M., Tognoni G. (1987). Standardizzazione e taratura italiana di test neuropsicologici. Ital. J. Neurol. Sci..

[32] Wilson Barbara A., Cockburn J., Halligan P.W., Spinazzola L., Pagliari C., Beschin N. (2010). BIT: Behavioural Inattention Test.

[33] Ratcliff G. (1979). Spatial thought, mental rotation and the right cerebral hemisphere. Neuropsychologia.

[34] Louis D.N., Perry A., Reifenberger G., von Deimling A., Figarella-Branger D., Cavenee W.K., Ohgaki H., Wiestler O.D., Kleihues P., Ellison D.W. (2016). The 2016 World Health Organization Classification of Tumors of the Central Nervous System: A summary. Acta Neuropathol..

[35] Tomaselli A., Luca A., Ferini G., Umana G.E., Chaurasia B., Scalia G. (2025). Cognitive Profiles and Determinants of Eligibility for Awake Surgery in Non-Dominant Hemisphere Gliomas: A Narrative Review. Brain Behav..

[36] Raffa G., Quattropani M.C., Marzano G., Curcio A., Rizzo V., Sebestyén G., Germanò A. (2021). Mapping and preserving the visuospatial network by repetitive nTMS and DTI tractography in patients with right parietal lobe tumors. Front. Oncol..

[37] Noll K.R., Ziu M., Weinberg J.S., Wefel J.S. (2016). Neurocognitive functioning in patients with glioma of the left and right temporal lobes. J. Neuro-Oncol..

[38] Young J.S., Morshed R.A., Andrews J.P., Cha S., Berger M.S. (2021). Prosopagnosia following nonlanguage dominant inferior temporal lobe low-grade glioma resection in which the inferior longitudinal fasciculus was disrupted preoperatively: Illustrative case. J. Neurosurg. Case Lessons.

[39] Cargnelutti E., Ius T., Skrap M., Tomasino B. (2020). What do we know about pre- and postoperative plasticity in patients with glioma? A review of neuroimaging and intraoperative mapping studies. Neuroimage Clin..

[40] Doherty C., Nowacki A.S., Pat McAndrews M., McDonald C.R., Reyes A., Kim M.S., Hamberger M., Najm I., Bingaman W., Jehi L. (2021). Predicting mood decline following temporal lobe epilepsy surgery in adults. Epilepsia.

[41] Van Sweden B. (1996). Sleep and the temporal lobe. Acta Neurol. Belg..

[42] Ayoub L.J., Barnett A., Leboucher A., Golosky M., McAndrews M.P., Seminowicz D.A., Moayedi M. (2019). The medial temporal lobe in nociception: A meta-analytic and functional connectivity study. Pain.

